# Changing schools in students with anorexia nervosa: escaping discredited identity

**DOI:** 10.1186/2050-2974-1-S1-P8

**Published:** 2013-11-14

**Authors:** Evelyn Bowtell, Rosalie Aroni, Julie Green, Susan Sawyer

**Affiliations:** 1 Centre for Adolescent Health, Royal Children's Hospital, Australia; 2 Department of Paediatrics, The University of Melbourne, Australia; 3 Murdoch Childrens Research Institute, Australia; 4 School of Public Health and Preventive Medicine, Faculty of Medicine, Nursing and Health Sciences, Monash University, Australia; 5 Royal Children's Hospital Education Institute, Australia; 6 Parenting Research Centre, East Melbourne, Australia

## 

Educational participation is central to adolescent peer relations, emotional wellbeing and future financial independence. The aim of this qualitative study was to explore the interface between the health and educational sectors to better understand how to support adolescents with chronic health conditions. Parents of adolescents with anorexia nervosa (AN, n=11), cancer (n=11) and cystic fibrosis (n=16) were recruited through two tertiary hospitals in Victoria, Australia. Audio-recorded in-depth interviews were conducted and transcribed verbatim. Just under half (5) of the AN parent cohort reported that their child changed schools during treatment due to identity concerns and a desire for a 'fresh start'; this was not apparent within the cancer or cystic fibrosis cohorts. Experience of stigmatisation was perceived as the major reason to change schools, which also appeared to explain why many parents did not inform the new school of the diagnosis. Efforts to avoid discredited social identity reduced opportunities for educational support as parents of students with AN had less opportunity, or less overt legitimate cause, to know about and access educational supports for children with chronic conditions than the other cohorts. In conclusion, the diagnosis of AN was frequently associated with school change and reduced opportunities for educational support.

